# Emotional stress as a trigger of falls leading to hip or pelvic fracture. Results from the ToFa study – a case-crossover study among elderly people in Stockholm, Sweden

**DOI:** 10.1186/1471-2318-9-7

**Published:** 2009-02-09

**Authors:** Jette Möller, Johan Hallqvist, Lucie Laflamme, Fredrik Mattsson, Sari Ponzer, Siv Sadigh, Karin Engström

**Affiliations:** 1 Karolinska Institutet, Department of Public Health Sciences, Division of Public Health Epidemiology, Stockholm, Sweden; 2 Uppsala University, Department of Public Health and Caring Sciences, Uppsala, Sweden; 3 Karolinska Institutet, Department of Public Health Sciences, Division of International Health, Stockholm, Sweden; 4 Karolinska Institutet, Department of Clinical Science and Education, Södersjukhuset, Section of Orthopaedic Surgery, Stockholm, Sweden

## Abstract

**Background:**

Sudden emotions may interfere with mechanisms for keeping balance among the elderly. The aim of this study is to analyse if emotional stress and specifically feelings of anger, sadness, worries, anxiety or stress, can trigger falls leading to hip or pelvic fracture among autonomous older people.

**Methods:**

The study applied the case-crossover design and was based on data gathered by face to face interviews carried out in Stockholm between November 2004 and January 2006 at the emergency wards of two hospitals. Cases (n = 137) were defined as persons aged 65 and older admitted for at least one night due to a fall-related hip or pelvic fracture (ICD10: S72 or S32) and meeting a series of selection criteria. Results are presented as relative risks with 95% confidence intervals.

**Results:**

There was an increased risk for fall and subsequent hip or pelvic fracture for up to one hour after emotional stress. For anger there was an increased relative risk of 12.2 (95% CI 2.7–54.7), for sadness of 5.7 (95% CI 1.1–28.7), and for stress 20.6 (95% CI 4.5–93.5) compared to periods with no such feelings.

**Conclusion:**

Emotional stress seems to have the potential to trigger falls and subsequent hip or pelvic fracture among autonomous older people. Further studies are needed to clarify how robust the findings are – as the number of exposed cases is small – and the mechanisms behind them – presumably balance and vision impairment in stress situation.

## Background

Falls leading to hip or pelvic fractures among older people is a large public health concern in many high income countries, not least in Sweden and in the other Nordic countries. In Sweden which has one of the oldest populations in the world, the life time risk (at 50 years) of a hip fracture is 28.5% among women and 13.1% among men [1]. Among those afflicted by hip fracture the majority are old (average age 81 years) and of female sex (75%) [2]. In Sweden, the majority of the people aged 65+ live alone in their own house or flat and about 15% of them have home-help in their dwelling or live in special service accommodation [3].

While age-related individual factors increase the vulnerability for fall-related injuries it has been established that environmental factors, both social and physical, also have an impact [4-6]. Among physical environmental factors, slippery floors, bad lightning and loose carpets are common direct reasons for falls among elderly people living at home [6]. Remote factors such as type of housing and marital status account for the social factors susceptible to increase the risk for hip fractures [5,7-9]. Likewise, environmental failures/mishaps, situations and events occurring shortly before a fall and subsequent injury are important dimensions to consider to better understand the causal chain leading to fractures [10,11]. Such factors, called triggers, influence both the occurrence of fall and the consequences of the fall in terms of injuries. In the present research project, called ToFa (Triggers of Fall), emotional stress has been studied as one potential trigger of fall-induced hip or pelvic fractures among the elderly.

Earlier studies, performed among both young people and adults, show that emotional stress influences the risk of being injured. In a study among children aged 10–15 we have established that different kinds of emotional states, caused by peer victimization as well as perceived school failure have the potential to trigger unintentional injuries [12-15]. Another study has shown that although anger is strongly related to intentional injuries among adult men and women it does not seem to have the same impact on fall injuries [16].

To the best of our knowledge, studies on the importance of different emotional stress and the risk for fall-related injuries and hip or pelvic fractures among elderly have not yet been published. The research on older people's emotions, so far, has focused on anxiety or fear of falling and how these factors affect the incidence of falls [17]. The aim of this study is to assess the trigger effects of emotional stress such as feelings of anger, sadness, worries, anxiety and stress, on the risk of falls and subsequent hip or pelvic fracture.

## Methods

To study triggers an epidemiological study design, called case-crossover, has proven useful [18]. The case-crossover design has been applied several times in order to study trigger factors of injuries. Alcohol, medication, intellectual exertion, and sleep deprivation are examples of factors that so far have been identified as triggers of different kinds of injuries [19-25].

The ToFa study is a case-crossover study designed to identify triggers of fall-induced hip or pelvic fractures. Cases were defined as patients admitted at least one night with a diagnosis of hip or pelvic fracture (ICD10: S72 or S32) and recruited at two emergency hospitals in Stockholm during November 2004 to January 2006. Screening of admissions to identify eligible cases was performed by one specially assigned research nurse at each hospital. Nine inclusion criteria had to be met. The patient should be (1) aged 65 years or above, (2) resident in Stockholm County, (3) Swedish-speaking, (4) living at home and not in an institution, (5) able to walk (with or without help of technical aids), (6) mentally healthy and free from addiction, (7) high scoring on the Short Portable Mental Status Questionnaire (SPMSQ) test of the cognitive capability: score ≥ 8 [26,27], (8) exposed to a recent low energy injury, and (9) without a pathologic fracture. Non-Swedish speaking patients (1.3%) were excluded to ensure high information quality in the interviews and cost effectiveness in data collection.

Participation was voluntary and written consent was compulsory. Totally 137 patients participated in the study, the majority (93%) diagnosed as ICD10 S72 (hip fracture) and seven percent with ICD10 S32 (pelvic fracture). Characteristics of the study population are shown in Table 1. The two hospitals have geographically defined catchment areas and together they treat about half of all hip fractures in the Stockholm region. During the study period the organization registered 881 patients, about half of the admitted cases (according to hospital register information). Fifty percent (440 patients) did not meet our inclusion criteria and of these, 40% did not pass the cognitive function test and 43% were too sick to participate. Among the 441 patients that did meet our inclusion criteria 33% were asked for participation. The patients not registered or not asked for participation were missed due to the work schedule and work load of the research nurses or participated in other studies. Among the patients asked, 79% agreed to participate. The 21% declining participation were similar to participants with regard to age and diagnosis, however a somewhat higher proportion was male (33%).

**Table 1:** Characteristics (%) of the study population in the ToFa study, Stockholm County, Sweden, 2004–2006, 137 patients.

Characteristic		Percentage

Gender	Men	21.9

	Women	78.1


Age	65–79	35.8

	80+	64.2


Birth country	Sweden	94.8


Civil status	Married	39.4


Housing type	House	17.5

	Apartment	75.2

	Flat in a block of service flats	5.1

	Other	2.2


Go out shopping		77.4


Eat at least 3 meals per day		73.7


Takes care of the home		70.8


Walks in stairs		62.0


Have home-help service		22.6


Get help to go out		16.1


Place of injury	Outdoor	32.3

	Indoor	67.6

A group of four research nurses were trained to conduct the interviews and also participated in regular meetings to discuss and solve arisen problems. The research nurses interviewed the patients to obtain exposure information and to establish the time of the injury. Ninety-three percent of the interviews were conducted within four days of the injury. The interview was structured and lasted approximately one hour. The questionnaire covered information about the injury, the timing of all main activities of the patient during the two days before the injury, exposure to potential triggers (48 hours prior to the injury and usual level of exposure frequency), general information on health status and health habits, individual information (such as sex, age, length, weight etc), and a score measuring the patient's fear of falling.

Emotional stress was defined as feelings of anger, sadness, worries, anxiety and stress and measured by the question: "Did it happen on the day of your injury or the day before that you felt *angry*?" If a patient answered 'yes' the patient was asked to specify exactly when they were angry during the 48 hours prior to the injury. Further, all patients were asked "How often during the last six months have you experienced feelings of anger?" The answer could be given as times per day/week/month or year, and was re-calculated to represent a usual annual frequency of exposure. The wording of the questions was similar for all the 5 different emotions.

A fall and subsequent injury as defined in this study can only occur during time spent walking, standing or in intermediate positions while changing posture. Accordingly, the person-time from which the control windows are sampled should ideally be restricted to times of outcome opportunity [18]. To correctly assess exposure frequencies during the person-time at risk of falling, every episode of walking, standing, sitting, lying and changing posture was thoroughly charted in the interview through a schedule of activities during the two days prior to the injury. To aid the ability to recall, the interviewer first ascertained usual daily activities and situations (for example time for getting out of bed, eating, medication intake, personal hygiene, visits, watching television, significant events etc). Information regarding the level of activity was retrieved for each 15-minute interval but smaller time intervals were registered in case the duration was shorter, such as a visit to the toilet.

### Statistical analyses

The risk estimate was calculated as the ratio between the observed odds of exposure to the expected odds of exposure [28]. The observed odds of exposure (either 1 or 0) were determined by the observed frequency of emotional stress during the hazard-period prior to the injury. Results are presented for hazard-periods of one hour. Hazard-periods of varying length were tested but the number of exposure events outside the one-hour interval prior to the injury was small, limiting the induction time analyses. The expected odds of exposure were defined as the proportion of exposed person-time to the proportion of unexposed person-time, and were assessed on basis of the recorded frequency of exposure during the two types of control periods preceding the fall, i.e., 48 hours or 6 months. In an exact calculation we based the expected odds of exposure on the complete person-time at risk (of falling) during the 24-hour period prior to injury (A in Table 2). Exposed time was determined from reported episodes of trigger exposure and calculated as the sum of the time at risk during each hazard-period following an episode of trigger exposure. Unexposed time was calculated as the total time at risk minus the exposed time. In cases experiencing emotional stress close to the injury this assessment of exposed time might lead to an underestimation of the exposed time since the last period of exposure sometimes was interrupted by the fall. In these rather few instances (n = 4) a more correct estimate of the exposed person time was obtained by adding the remaining time at risk at the same point in time the day before.

**Table 2:** Relative risk (95% CI) of fall-induced hip or pelvic fracture within 1 hour after emotional stress (n = 122).

			Emotion		
Analytic strategy	Expected odds based on		Anger	Sadness	Stress

	Time at risk	Exposed time	Number of exposed cases = 4	Number of exposed cases = 2	Number of exposed case = 6

A	Standing/walking time^1^	Exact calculation^5^	12.2 (2.7–54.7)	5.7 (1.1–28.7)	20.6 (4.5–93.5)

B	All^2^	Usual frequency^6^	22.5 (7.4–68.8)	3.3 (0.7–15.0)	15.1 (3.8–59.8)

C	Time awake^3^	Usual frequency^6^	14.3 (4.7–44.0)	2.4 (0.5–10.8)	8.8 (2.3–34.6)

D	Standing/walking^4^	Usual frequency^6^	3.9 (1.2–12.7)	0.8 (0.2–3.2)	3.7 (1.0–13.8)

E	Standing/walking^4^	Usual frequency corrected for time at risk^7^	14.3 (4.7–44.0)	2.4 (0.5–10.8)	8.8 (2.3–34.6)

Five different analytic strategies varying the bases for the expected odds.

^1 ^Time spent walking or standing during the 24 hour period prior to injury

^2 ^Total time per year (8760 hours)

^3 ^Time awake per year (estimated from time from going to bed until waking up)

^4 ^Yearly time spent walking or standing (estimated from time spent walking or standing during the 48 hour period prior to injury)

^5 ^Sum of the time at risk during each hazard period following an episode of trigger exposure. in the 24 hour period prior to injury.

^6 ^Annual frequency of exposure

^7 ^Corrected annual frequency of exposure under the assumption that exposure occurs independently of time at risk, correction is made of the frequency of exposure to correspond to the proportion of time at risk (of time awake).

To examine the robustness of the exact calculation, we also calculated the expected odds of exposure using self-reported usual frequency of trigger exposure according to the standard case-crossover methodology and applied three different estimations of the time at risk leading to four alternative analytical strategies (B-E in Table 2) [18]. Person-time at risk of falling was defined as (B) the total time during a year equal to 8760 hours, (C) the total time minus individual sleeping time measured by the time between going to bed and waking multiplied by 365, and (D+E) as an average time at risk per year calculated from the proportion of time spent standing or walking during the 48 hours prior to the injury divided by 2 and multiplied by 365. Exposed person-time was estimated as usual annual frequency multiplied with the assumed length of the hazard period (B, C, D) implicitly making the conservative assumption that all trigger exposures occurred during time at risk. In (E) we instead explicitly assumed that trigger exposure occurred independently of whether the person was at risk of falling or not. Hence, exposed person-time was proportionally reduced. In all these calculations the trigger exposures were assumed to be discrete and their effect periods not to overlap.

The effect estimates were calculated as standard Mantel-Haenszels estimates with confidence intervals for sparse data [18,29]. Results are presented as relative risks and with 95% confidence intervals. All calculations were performed using the SAS software package version 8.02.

The study was approved by the Regional Ethics Committee in Stockholm.

## Results

Thirty three of the 137 patients (24.1%) experienced at least one of the emotions during the 24 hour period prior to injury. Among these there were 15 who reported that they had experienced the emotions during the day of the injury but could not state any specific episode with an exact timing, in most cases because the emotion lasted all day. They were excluded from further analyses.

During the hazard period of one hour before the fall 3.3% (4/122) of the cases were exposed to anger, 1.6% (2/122) to sadness and 4.9% (6/122) to stress, in all 10 patients. Only one of the patients experienced more than one type of emotion during the hazard period. The number of cases exposed to feelings of worry and anxiety during the hazard period was too small to be further analysed.

The analysis based on a complete follow-up of exposed and unexposed person-time during the 24 hours prior to injury (A column in Table 2) shows that the increased relative risk of fall-related hip or pelvic fracture during the first hour after emotional stress is 12.2 (95% CI 2.7–54.7) for anger, 5.7 (95% CI 1.1–28.7) for sadness and 20.6 (95% CI 4.5–93.5) for stress.

The other analytical strategies confirm the results although the risk estimates change slightly. Analysis B, does not take into account that the risk of a fall and subsequent injury is only present when standing, walking or changing posture. Further, the proportion of exposed follow-up time at risk is obtained through the question of usual frequency multiplied with the hazard period. It resulted in relative risks of 22.5 (95% CI 7.4–68.8) for anger and 15.1 (95% CI 3.8–59.8) for stress. If the expected odds of trigger exposure are independent of whether the case is at risk of falling or not the effect estimate would be the same. The slightly higher RR in B than in A suggests that the expected odds of exposure are underestimated in B due to an overestimation of the proportion of unexposed time at risk. In analysis C this problem is reduced by restricting the study base to time awake, correctly assuming that no falls happen while asleep, and that there is also no conscious emotional stress while sleeping. Accordingly, the relative risk estimates were reduced to 14.3 (95% CI 4.7–68.8) for anger and 8.8 (95% CI 2.3–34.6) for stress.

In the results presented in the last two rows, the study base time was restricted to the time at risk estimated by the individual, self-reported time at risk of falling during the 48 hours before the fall. In analysis D we assumed that all emotional stress events in the self-reported usual frequency occur during time at risk of falling. As some of the anger, sadness or stress may have happened during time spent sitting or lying we consciously overestimated the exposed time implying an underestimation of the relative risk [18,28]. Still, these analyses showed elevated risk estimates, 3.9 (95% CI 1.2–12.7) for anger and 3.7 (95% CI 1.0–13.8) for stress. In analysis E we instead assumed independence between emotional events and the risk of falling implying that a proportionate number of emotional events take place during time at risk and during time not at risk. How many of the exposure events that should be assumed to take place during time at risk was decided by the proportion of time at risk of the total time awake. For example, a patient reporting standing or walking approximately 3 hours per day would be at risk of falling 20 percent of the time (given that 9 hours of sleeping time is excluded). If the same patient reported anger occurring five times per day the reported frequency of emotional stress will be reduced to 20%, namely to once a day. Analysis E resulted in elevated relative risk of 14.3 (95% CI 4.7–44.0) for anger and 8.8 (95% CI 2.3–34.6) for stress, similar to the effect estimates reported for analysis A.

## Discussion

This study shows that, among autonomous elderly, emotional stress may trigger falls leading to hip or pelvic fractures. The results reveal an increased risk in the period of one hour after anger or perceived stress. This is the first study to report a trigger effect of emotional stress on the risk for fall and subsequent hip or pelvic fracture. In a combined case-control and case-crossover study Vinson et al found an association between state anger and risk of injury. In sub-analyses of different types of injury they reported increased risk estimates for fall injuries however statistically non-significant [16].

There are two possible and closely related mechanisms that may explain the relationship between emotional stress and injurious fall: impoverished postural control and gaze strategy. Aging is frequently accompanied by deterioration in postural control (and preparation) and it has been observed that in order to maintain balance, age-specific compensatory strategies involving the hip (rather than the ankle as in younger subjects) and local muscles (thigh ones) are used to counterbalance a decrease in anticipation [30]. Further, age-related deficits in the neuro-musculoskeletal systems may impede ability to effectively execute "change-in-support" (CIS) balance-recovery reactions that involve rapid stepping or reaching movements that play a critical role in preventing falls [31].

In some groups of frail older people (e.g., women aged 75–86 years with low bone mass), falls-related self-efficacy may be independently associated with both balance and mobility after accounting for age, current physical activity level, and performances in relevant physiological domains [32].

The changing gaze strategy is related to poor vision. It is known that poor visual acuity reduces postural stability and significantly increases the risk of falls and fractures in older people [33]. It is also possible that older people, in stress situation would adopt a gaze strategy detrimental to their balance control, more specifically, a premature gaze transfer which in turn has been associated with decline in stepping accuracy and precision [34]. Studies that have included multiple visual measures have found that reduced contrast sensitivity and depth perception are the most important visual risk factors for falls [34].

### Methodological considerations

A peculiar feature of studies of falls is that person time at risk is not continuous in real time. The fact that there is only an outcome opportunity when walking, standing, sitting or changing posture calls for special measures when applying the case-crossover design. Measuring information of the time at risk is not easy and many studies do not have the possibility to control for it when calculating the effect estimates. In this study we made an effort to collect such information in addition to traditional information for making case-crossover analyses. Therefore we had the opportunity to test different analytic strategies and the robustness of the found effects. Each analytic strategy implies, however, different degrees and aspects of methodological problems.

The first analysis (A) we performed is as close to correct as we think is possible. The analysis was based on an exact calculation of exposed time thoroughly controlled for time at risk. The narrow time interval of 24 hours was chosen to minimize the risk for information bias and to be able to impute risk time information for exposed cases. Ignoring this could lead to a small underestimation of the exposed time among the exposed cases which in turn could lead to an overestimation of the relative risk.

Had we not had information regarding this time at risk we would have been limited to basing the exposed time on the reported usual frequency of trigger exposure. Besides any information bias in such a variable, the analysis based on this has the disadvantage that the reported exposure information is not restricted to times at risk. For this reason we tested to restrict the study base time in three different ways. Analysis B is a straight forward case-crossover analysis falsely assuming that the risk of falling is equal over the whole day. This leads to an overestimation of the proportion of unexposed time and an overestimation of the relative risk. Analysis C restricted the study base time to time spent awake. This reduces the overestimation of the unexposed time but still, depending on the exposure, leads to an overestimation of the relative risk. Analyses D and E was based on the reported person time at risk. The methodological objection to these analyses is due to the fact that the reported usual trigger frequency was not restricted to time at risk. Making assumptions regarding the distribution of exposure over time at risk and time not at risk is difficult. In analysis D, we made the conservative assumption that all episodes of emotional stress occurred during time at risk. Under the premise that it is unlikely that emotional stress always takes place during time at risk, this analysis leads to an overestimation of the exposed time leading to underestimated effect estimates. In analysis E, the overestimation of exposed time was reduced as the frequency of exposure was distributed correspondingly to the distribution of time at risk to time not at risk. However, this assumption might for some individuals not be true, for example if a person becoming angry always starts to move around in order to physically get rid of the anger. On the other hand, if a patient becoming angry always tries to calm down by sitting down this would also be an incorrect assumption. To test this further, additional information has to be measured on an individual level, which we prompt future studies to take into account if not using the exact method.

The restriction to patients with a normal cognitive function and in some analyses to an exposure period of 24 hour or 48 hour prior to the injury, together with an interview after as little delay as possible, would diminish recall problems leading to non-differential misclassification. To further improve recall of exposures, the interview charted activities during the two days prior to the event and there were good possibilities to link the fall and also potential triggers to activities, events and things that had happened during this time period. According to information from the research nurses carrying out the interviews, this group of elderly people lives organized lives with little variations in their daily activities which further aid their memory. Only a small number of patients reported that the 48 hour period prior to injury was "an unusual day". Excluding those from the analysis did not alter our conclusions. It is also reassuring that analyses based on short and long recall periods show similar results.

Additionally, to combat differential misclassification of exposure between the case and control period, neither the patients nor the interviewers were aware of the assumptions regarding the induction times and were instructed to pay equal interest to the whole 48-hour period, hence, an attribution of exposures in those periods seems unlikely. As hip or pelvic fracture is an acute event, exposure in the case period might otherwise be less likely to be missed or forgotten.

Instances of emotional stress were assessed by simple interview questions allowing for a dichotomous classification. No intensity scales were used. Because the case information is compared with control information from the same patient, there will be no differential exposure misclassification as long as each individual patient uses the same definition of every exposure in all questions, which seems a reasonable assumption. Exposure to other triggers of falls in the period prior to onset might imply confounding. However, co-exposure between the five different emotions was uncommon in this study. Only three patients were exposed to two or three emotions simultaneously during the three-hour period prior to the injury. Exclusion of these cases in the analyses decreased power but did not alter the direction of the estimated effects.

Non-participation because the research nurses did not manage to contact all cases would foremost be a result of the nurses' work load and working hours and not selective with regard to the case's exposure status in the case period. The patients were not informed about the questions in the interview and unaware of the fact that emotional stress would be covered. Hence, it is unlikely that exposure status would have affected their participation. Survival bias is no problem as mortality in the early period after a hip or pelvic fracture is low. The restriction in the recruitment of all patients with a hip or pelvic fracture caused by the inclusion criteria does not lead to bias.

If the patients have difficulties in differentiating between emotions prior to the injury and emotions arising due to or post the injury, it would imply bias. However, of the ToFa patients reporting emotions prior to the injury (in the case window) only one reported it to have happened directly in relation to the fall. The rest of the patients reported it to happen longer before the event (such as 25 minutes) which most likely cannot be mistaken for the feelings arising due to the fall.

A further limitation of the study is the small number of exposed cases. Although the effects are statistically significant, the confidence intervals are wide and this challenges the robustness of the estimated effects.

## Conclusion

Emotional stress seems to have the potential to trigger falls and subsequent hip or pelvic fracture among autonomous older people. However, to our knowledge, this is the first study to report this and further studies are needed to clarify how robust those findings can be and disentangle the mechanisms behind these findings, likely to be related to balance and vision impairment in stress situation.

## Competing interests

The authors declare that they have no competing interests.

## Authors' contributions

JM contributed to the conception and design of the ToFa study, made the statistical analyses and interpretation of the data, and drafted the manuscript. JH contributed to the conception and design of the ToFa study, interpretation of data, and drafting of the manuscript. LL conceived the ToFa study and contributed to the conception, design, acquisition of data, and interpretation of data, and drafting of the manuscript. KE contributed to the conception, design, and acquisition of data and have revised the manuscript for important intellectual content. FM participated in the statistical analyses and interpretation of the data and has revised the manuscript for important intellectual content. SP made substantial contributions to acquisition of data and has revised the manuscript for important intellectual content. SS made substantial contributions to acquisition of data and has revised the manuscript for important intellectual content. All authors have read and approved the final manuscript.

## Pre-publication history

The pre-publication history for this paper can be accessed here:


